# Room-temperature ethylene glycol sensor based on cuprous oxide/MXene films

**DOI:** 10.1038/s41598-025-12019-1

**Published:** 2025-08-05

**Authors:** Sepehr Samiei, Asadollah Kalantarian, Azam Iraji zad, Narges Darmiani

**Affiliations:** 1 Center for Nanoscience and Nanotechnology, Institute for Convergence Science and Technology, Sharif University of Technology, Tehran, 14588-89694 Iran; 2 Department of Physics, Sharif University of Technology, Azadi Street, Tehran, 11365-9161 Iran

**Keywords:** MXene, Cu_2_O/MXene bilayer, Quartz crystal microbalance (QCM), Gas sensors, Ethylene glycol (EG), Nanoscale materials, Nanoscale materials, Sensors

## Abstract

This study demonstrates a high-performance room-temperature ethylene glycol (EG) gas sensor using Cu_2_O/MXene bilayer films on quartz crystal microbalance (QCM) substrates, addressing critical needs for industrial safety and environmental monitoring. The fabricated sensors were systematically characterized by XRD, FTIR, and FESEM, revealing that the Cu_2_O/MXene bilayer configuration achieved exceptional performance with an ultra-low detection limit of 381 ppb, high sensitivity of 22.8 Hz/ppm, and excellent selectivity compared to individual Cu_2_O, MXene, or their mixture films. The enhanced sensing capability originates from synergistic effects between p-type Cu_2_O and conductive MXene, forming a Schottky junction that facilitates charge transfer and promotes EG adsorption through combined physisorption mechanisms involving hydrogen bonding with MXene’s functional groups (OH, O, F) and interactions with oxygen species on Cu_2_O nanoparticles. At 72 ppm EG concentration, the bilayer sensor exhibited 12.6-fold, 3.6-fold, and 2.34-fold higher response than pure Cu_2_O, MXene alone, and their mixture film, respectively. While humidity tests showed a moderate ~ 15% response reduction at 60% RH, the Cu_2_O/MXene bilayer maintained robust performance, establishing it as a cost-effective and reliable room-temperature sensing platform suitable for next-generation gas detection applications in challenging environments.

## Introduction

Unprecedented industrial growth in recent decades, though instrumental in economic development and lifestyle improvements, has concurrently generated massive increases in emissions of noxious and dangerously reactive gases. These emissions not only degrade air quality but also compromise public safety and pose severe risks to human health. As a result, gas sensors have become indispensable in applications ranging from environmental monitoring and industrial safety to national defense, disease diagnosis, and food inspection^1–5^.

Ethylene glycol (HOCH_2_CH_2_OH), a vital colorless and sweet-tasting solvent widely utilized in industries for antifreeze, coolants, and polyester production, presents substantial health hazards. Its toxic fumes, particularly when exposed to high temperatures (80–90℃), can be harmful^6^. Its hydroxyl group oxidizes into glycolic acid and oxalic acids in the human body, causing severe health issues such as respiratory distress, pulmonary edema, and detrimental effects on the nervous system, heart, and even fatality^7^. In the liver, ethylene glycol (EG) is initially converted into glycoaldehyde by alcohol dehydrogenase, and subsequently transformed into a series of toxic metabolites. The formation of insoluble calcium oxalate due to oxalic acid accumulation can cause renal damage, while the production of formic acid may lead to blindness. To safeguard public health, the American Academy of Clinical Toxicology has defined strict exposure thresholds, including a permissible concentration time-weighted average (PC-TWA) of 7.2 ppm and short-term exposure limit (PC-STEL) of 14.4 ppm^8^. Achieving reliable detection of EG at room temperature with high specificity remains a significant challenge in sensor technology.

Recent studies have aimed to improve EG sensor performance. As summarized in Table 1, for instance, Wang et al. developed a NiO foam@Sn-doped In_2_O_3_ nanowire sensor that exhibited a high response at 125 °C^9^, while Niavol et al. reported mesoporous Zn_2_SnO_4_ nanostructures operating at 270 °C^10^. Despite these advances, the high operational temperatures and power requirements of these sensors limit their practical deployment, particularly in settings where energy efficiency, sensor longevity, or cost are critical.

Most reported EG gas sensors are of the resistive type, which often suffer from temperature-control issues and high power consumption. At temperatures above 80℃, EG is readily oxidized into glycolaldehyde (HOCH_2_CHO) and other by-products, reducing sensor specificity. By lowering the operating temperature, unwanted chemical reactions can be minimized, thereby improving both the accuracy and efficiency of the sensor system. This approach paves the way for the development of cost-effective and reliable sensors suitable for a broad range of industrial applications.

Among various room-temperature gas sensor technologies, Quartz Crystal Microbalance (QCM) sensors have attracted significant attention due to rather simple electronic circuitry and the ease with which their surfaces can be modified^11–13^. QCM sensors operate on the principle that the resonant frequency of a quartz crystal changes in response to mass loading^14^. This capability allows QCM sensors to detect nanogram-level changes in mass, enabling highly sensitive and selective detection of a wide range of gases^15,16^. The Sauerbrey equation provides the fundamental quantitative relationship between resonant frequency shifts and adsorbed mass changes in piezoelectric systems^17^:

1 $$\:\varDelta\:{f}_{n}=\:-n\frac{2{f}_{0}^{2}}{\sqrt{{\mu\:}_{q}{\rho\:}_{q}}}{\varDelta\:m}_{a}$$

Where $\:\varDelta\:{f}_{n}$ is the change in resonant frequency at the n^th^ harmonic (Hz), $\:n$ is the harmonic order, $\:{f}_{0}$ is the resonant frequency (Hz), $\:{{\upmu\:}}_{q}$ is the shear modulus of quartz (Pa), $\:{{\uprho\:}}_{q}$ is the density of quartz in (kg/m^3^), and $\:{\varDelta\:m}_{a}$ is the areal mass of the film (kg/m^2^).

Despite these advantages, bare QCM sensors typically show limited response to gas molecules owing to insufficient physisorption interactions between the gas and the sensor surface. To enhance their performance, various surface modification strategies have been employed. These include the use of nanostructured 2D materials^16,18^, porous semiconductor metal oxides and polymers^19–23^ as well as hybrid materials^24–27^.

MXenes, are a prominent class of 2D materials renowned for their exceptional physical, chemical, and electrical properties, including favorable chemical stability, biocompatibility, and optical properties^28,29^. These inherent characteristics make MXenes highly appealing for advanced applications in catalysis, energy storage, and sensor devices^30^. In gas sensing, MXenes have demonstrated remarkable detection capabilities for target analytes such as nitrogen dioxide (NO2), ammonia (NH3), and hydrogen (H2)^31–35^. This performance originates from their chemically active surface terminations (-OH, -F, -O), which induce a net negative surface charge density and contribute to enhanced gas adsorption and sensitivity. To optimize sensing performance, particularly sensitivity and selectivity, MXenes are often combined with other nanomaterials to form heterostructures. For example, Liua et al. synthesized ZnO/Ti_3_C_2_T_x_ nanocomposites and performed resistive NO_2_ sensing. The composite samples exhibited higher sensitivity than pure ZnO films^36^.

Cuprous oxide (Cu_2_O), a p-type semiconductor with applications in photocatalysis and gas sensing, represents another promising material for synergy with MXene^37^. Previous studies have shown that layer-by-layer self-assembly (LBL-SA) of Cu_2_O nanoparticles with graphene oxide (GO) can be effectively used for detecting trimethylamine (TMA) via QCM at room temperature. The integration of GO and Cu_2_O in a nanocomposite structure leads to enhanced physical adsorption capabilities attributed to two main factors: the enlarged surface area and the formation of p-n junctions^38^.

This work demonstrates the development of Cu_2_O/MXene bilayer films on QCM substrates for room-temperature EG detection. Our data indicates that the QCM technique represents an effective method for EG detection with a molecular weight of 62.07 g/mol. The sensor films were characterized using X-ray diffraction (XRD), scanning electron microscopy (SEM), and Fourier Transform Infrared Spectroscopy (FTIR). Frequency shifts of the samples were determined for room temperature exposure to gas concentrations above and below the trace-level detection (TLD) limit. The gas-sensitive performance of the Cu_2_O/MXene bilayer sensors was evaluated and compared with MXene, Cu_2_O, and their mixture samples. Results on the bilayer exhibited a synergetic effect of Cu_2_O with MXene and a relatively good performance. Furthermore, this study investigated the influence of humidity on the sensor’s performance characteristics.

Frequency shifts were measured at room-temperature for EG concentrations above and below the trace-level detection (TLD) limit, demonstrating that the Cu_2_O/MXene bilayer exhibits a synergistic effect that markedly enhances gas sensitivity relative to individual Cu_2_O, MXene films, and their mixtures. Furthermore, the influence of ambient humidity on sensor performance was investigated. Our findings indicate that the QCM-based sensor platform, operating at room temperature, offers a promising route to low-cost, energy-efficient EG detection for environmental surveillance, industrial hazard prevention, and health protection systems.

## Experimental section

### Materials and equipment

All reagents employed in this investigation were of analytical purity and utilized without additional purification. Cuprous oxide (Cu_2_O, CAS No.: 1317-39-1) and the Ti3AlC2 MAX phase (CAS No.: 196506-01-1) were sourced from Sigma Aldrich. Hydrochloric acid (HCl, 98%) was obtained from Merck & Co., Quartz crystals (10 MHz, AT-Cut mode) coated with silver were acquired from Abracon Co., and the associated crystal oscillator was sourced from Novaetech Co. (OpenQCM store).

### Synthesis and characterization

MXene was synthesized in our previous work as reported by Darmiani et al.^30^. As such, a selective etching process was employed to remove the aluminum layers from the purchased Ti_3_AlC_2_ MAX phase. First, an etching solution was prepared by dissolving 1.6 g of lithium fluoride (LiF) in 20 mL of 9.0 M HCl under continuous stirring for 10 min. Next, 1 gram of Ti_3_AlC_2_ powder was incrementally added to the solution, which was then held at 40 °C for 40 h. Following the reaction completion, the product underwent extensive washing before being resuspended in DI water. The resulting suspension was subjected to argon-protected sonication followed by centrifugation (3500 rpm, 1 h), yielding a dark green supernatant that was meticulously harvested for further characterization.

To characterize the synthesized MXene, Fourier Transform Infrared Attenuated Total Reflectance (FTIR-ATR) spectra were acquired across the mid-infrared region (400–4000 cm^− 1^) employing a 4 cm^− 1^ resolution. It was verified that our synthesized MXene showed similar spectra as Darmiani et al.^30^.

The characterization of the synthesized MXene/Cu_2_O films, deposited on QCM chips, was performed using several techniques. The structural and morphological properties were examined using a TESCAN-Mira III field-emission scanning electron microscope (FE-SEM) operated at an acceleration voltage of 5 kV.

### Preparation of the sensing films

Quartz crystal sensors were cleaned by sonication in acetone and DI water for 5 min each. To prepare the Cu_2_O/MXene bilayers, 10 µ‏L of ultrasonicated MXene solution (6.5 mg/mL) was drop-casted into the center of the electrode and dried at 80 °C for 10 min. Subsequently, 10 µ‏L of Cu_2_O particles dispersed in ethanol with various concentrations (varying from 0.5 to 6 mg/mL) were drop-casted onto the electrodes. For mixture films, Cu_2_O particles were mixed with MXene solution at the same concentrations and similarly applied on the electrodes. The morphology and thickness of the films were analyzed using FTIR and SEM.

### Analyte detection using QCM technique

Various volatile analytes including EG, deionized (DI) water, isopropyl alcohol (IPA), formaldehyde (HCHO), ethanol, acetone, and methanol were tested. All analytes had purities above 99%. Gas response measurements were performed in a sealed 4-liter chamber containing ambient air. Liquid analytes were injected onto a small heated glass Petri dish placed inside the chamber, facilitating their evaporation and thorough mixing with the air. The chamber was equipped with a fan to ensure uniform distribution of the target gases. The methodology for calculating the gas concentration of each analyte is detailed in the following steps (Eqs. (2)-(5))^39^.

1. The moles of the injected analytes are calculated using Eq. (2):

2 $$\:n=\frac{mass\:of\:analyte}{{M}_{w}}=\frac{\rho\:\times\:v\times\:x}{{M}_{w}}$$

Where, $\:{M}_{w}$ represents the molar mass of the analyte in g/mol, $\:{\uprho\:}$ denotes the analyte density in g/mL, $\:v$ is the volume of the liquid analyte in mL, and $\:x$ signifies the purity of the analyte as a dimensionless fraction (e.g., 99% purity is expressed as 0.99).

2. The gas volume of our analyte after evaporation can be obtained using Eq. (3):

3 $$\:{V}_{analyte}=n\times\:{V}_{m}$$

Where, $\:{V}_{m}\:$is the molar gas volume in L/mol at temperature $\:T.$

$3.  \:{V}_{m}$ can be obtained from Eq. (4), which is derived from the Ideal Gas Law assuming constant atmospheric pressure:

4 $$\:{V}_{m}={V}_{m0}\times\:\frac{T}{{T}_{0}}$$

Where, $\:{T}_{0}$​ = 273.15 K (0 °C) is standard temperature, $\:T\:$= 298.15 K (25 °C) is our operating temperature, and $\:{V}_{m0}$ is the molar gas volume at standard temperature $\:{T}_{0}$ and is equal to 22.414 L/mol.

4. And finally, the analyte gas concentration in ppm is thus given by Eq. (5):

5 $$\:c=\left(\frac{{V}_{analyte}}{V}\right)\times\:{10}^{6}=\frac{{V}_{m}\times\:{\uprho\:}\times\:v\times\:x\times\:{10}^{6}}{{M}_{w}\times\:V}$$

Where, V is the chamber volume in L.

**Fig. 1:**
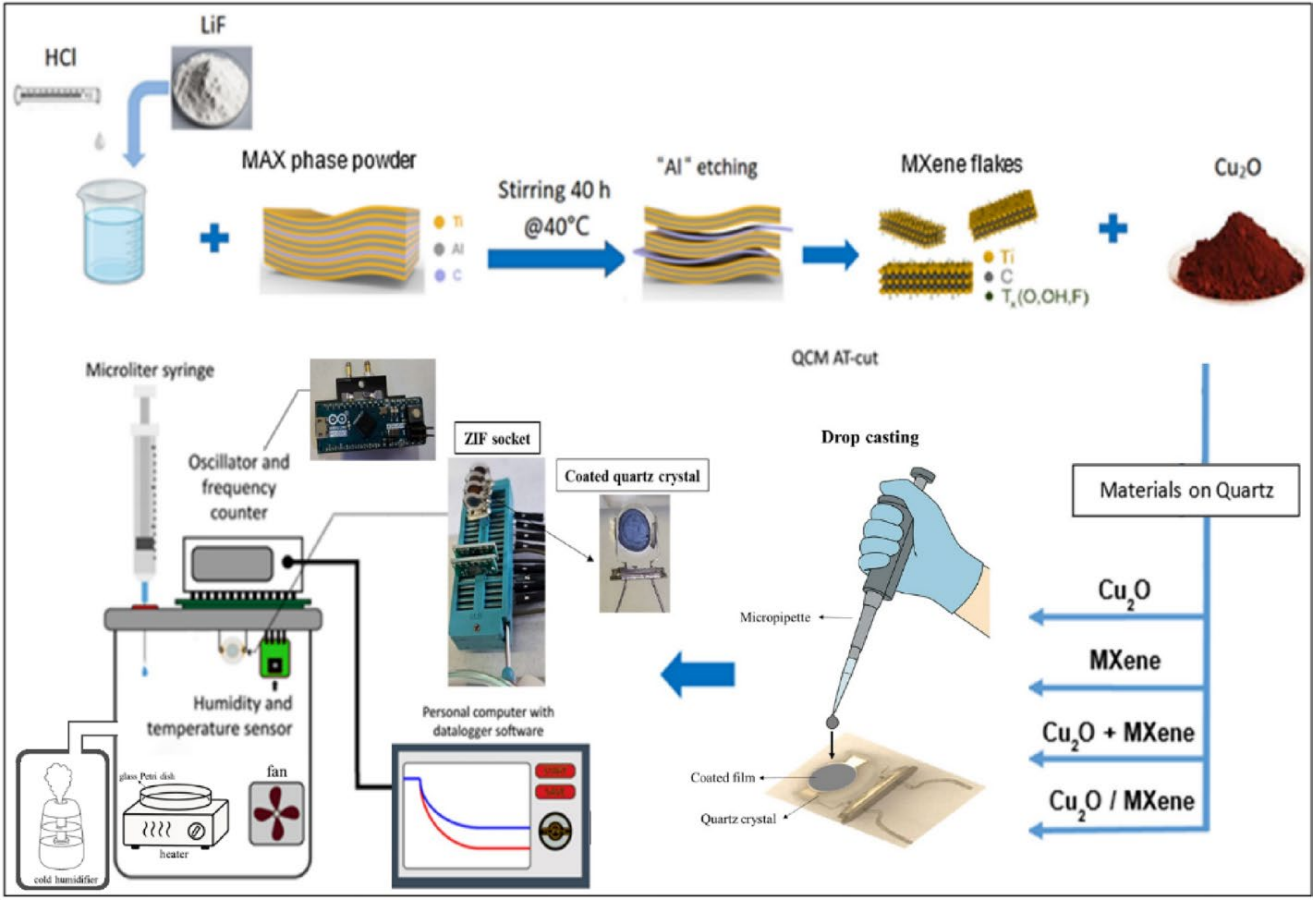
Integrated schematic and optical view of the QCM-based sensor constructed using drop-casted films.

The chamber conditions were 30% relative humidity and 25 ± 5 °C. The resonant frequency shift caused by exposure to the analyte in the air chamber was measured three times for each analyte at room temperature. To study the effects of humidity, the relative humidity (RH) inside the sensing chamber was controlled using an ultrasonic cold humidifier connected to the chamber and a RH sensor placed nearby the sample. The humidifier generated water vapor until the RH reached a preset level, at which point it automatically switched off. For gas exposure, a known volume of EG was introduced into the chamber using a micropipette onto a cleaned glass dish placed above a heater to facilitate controlled evaporation, ensuring a stable concentration of EG vapor. Tests were conducted at ∼25 °C with RH varying from 30 to 99%. Fig. 1 provides an integrated schematic and optical view of the sensor structure and experiment setup.

## Results and discussion

### Characterization of the sensing films

The successful synthesis of Ti_3_C_2_, along with its negative zeta potential and X-ray diffraction (XRD) results, were thoroughly investigated and reported in our previous study^30^.

**Fig. 2:**
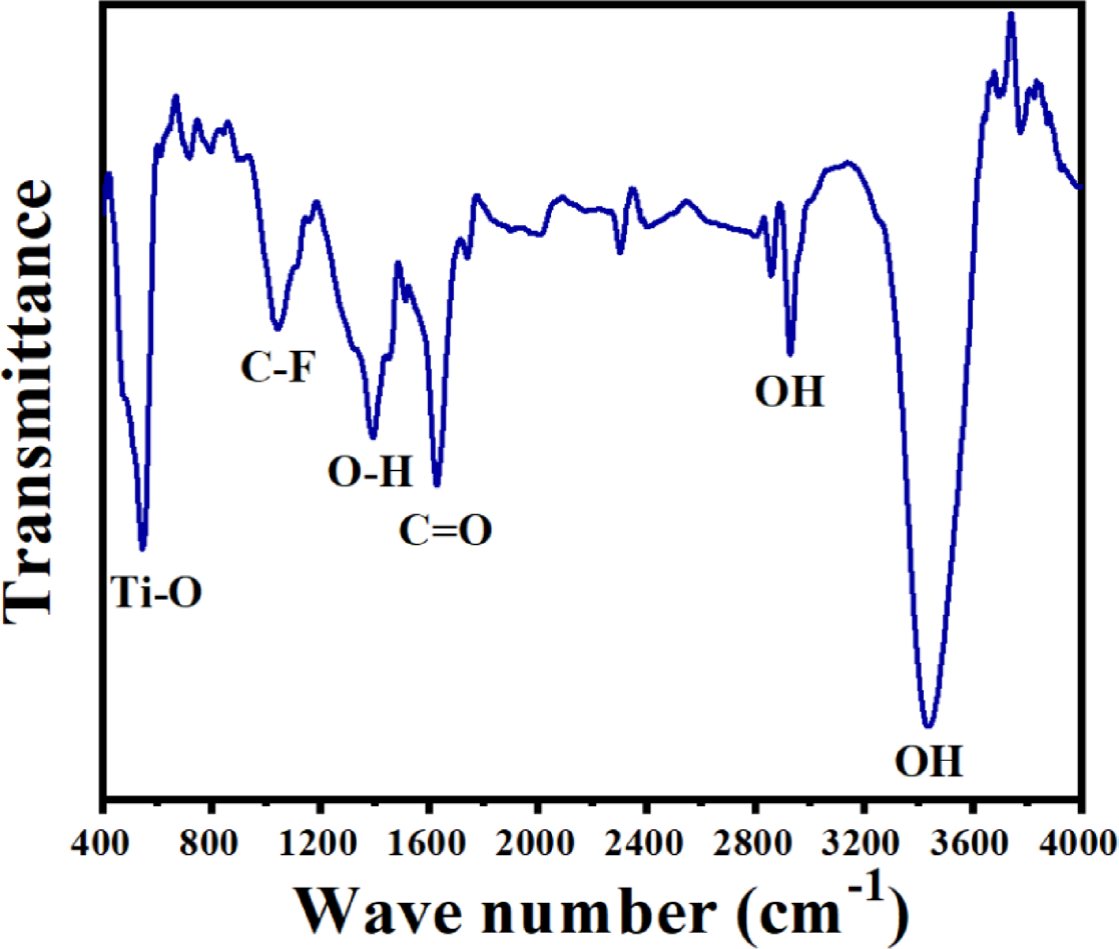
FTIR spectrum of the synthesized MXene.

Surface functional groups on MXene were identified using FTIR-ATR spectroscopy, as illustrated in Fig. 2. The FTIR spectrum exhibited absorption peaks at 3427 cm^−1^ and 2925 cm^−1^, characteristic of hydroxyl groups associated with adsorbed and coordinated water molecules^40–42^. Additionally, a peak at 1627 cm^−1^ indicated the presence of C = O bonds, while peaks at 1381 cm^−1^ and 1095 cm^−1^ were attributed to molecular water^41,43^ and C–F stretching vibrations^43^, respectively. A deformation vibration corresponding to the Ti–O bond was observed at 547 cm^−1^, confirming the presence of oxygen-containing functional groups on the surface^41–44^. These identified functional groups (–OH, –F, –O) suggest that the Ti_3_C_2_ surface is terminated with negatively charged moieties, a finding further supported by zeta potential measurements^30^. Consequently, the hydrophilic properties imparted by these functional groups enhance the material’s effectiveness in sensing applications.

**Fig. 3:**
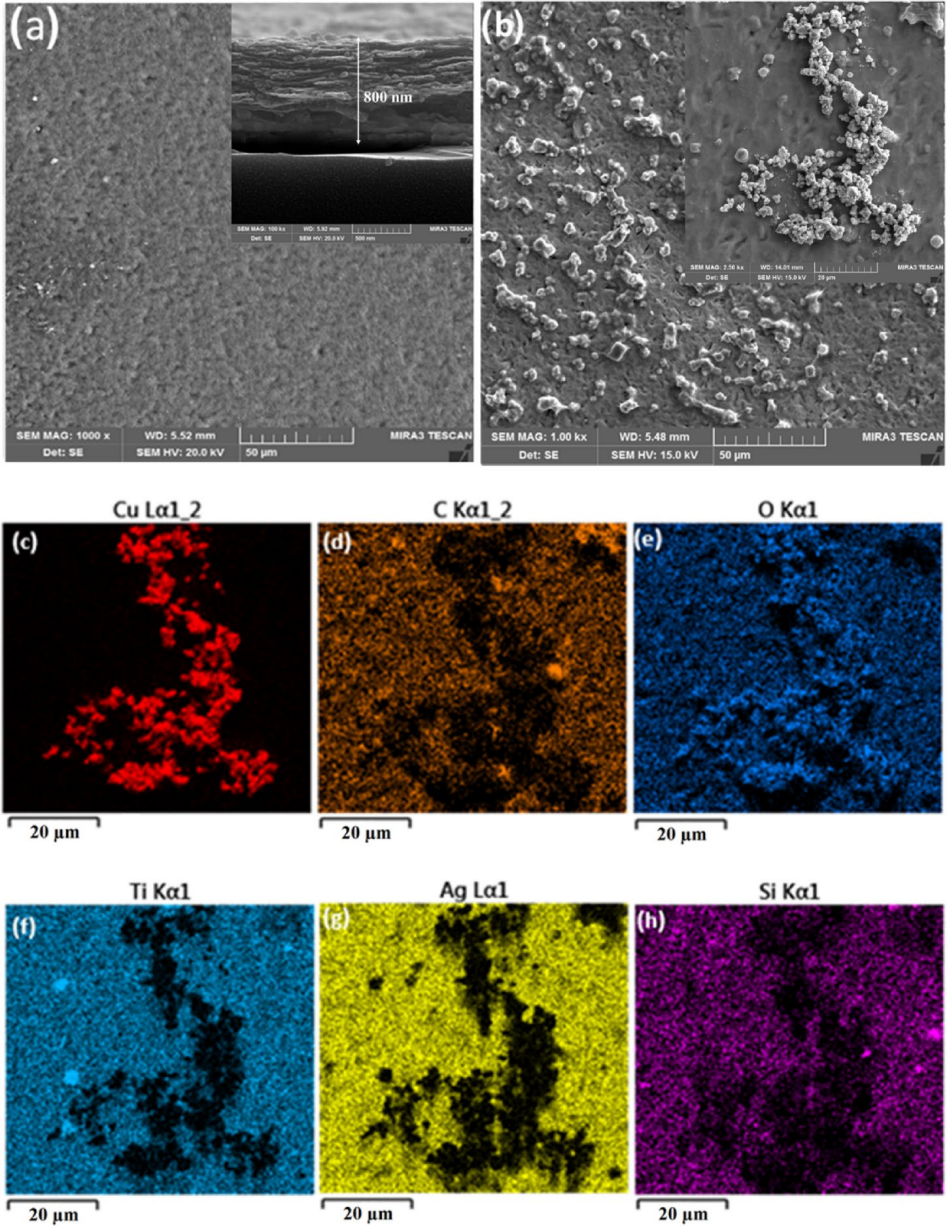
FESEM images of the prepared samples. (**a**) MXene film on Ag electrode and its cross-section (**b**) Cu_2_O particles on MXene substrate c-h) Elemental mapping of Cu_2_O/MXene bilayer from EDS.

The morphologies of the Cu_2_O, MXene, and Cu_2_O/MXene samples were analyzed using Field Emission Scanning Electron Microscopy (FESEM). Fig. 3a and its inset displays tightly packed MXene nanosheets on the quartz substrate, with a thickness of about 800 nm. Clusters of micron-sized crystals were observed on the MXene surface as depicted in Fig. 3b and its inset. To further analyze the composition of these clusters, Energy Dispersive X-ray Spectroscopy (EDS) was employed confirming the composition and distribution of the carbon (C), titanium (Ti), oxygen (O), and copper (Cu) elements, indicating the clustering of Cu_2_O on the MXene surface, as shown in Figs. 3c-h.

### Sensing properties of MXene-based QCM sensors

To evaluate the performance of different sensor configurations, we prepared four types of samples: one with a single-material coating of Cu_2_O particles, one with a single-material coating of MXene sheets, one with a mixture of both, and one with Cu_2_O deposited on MXene. Using 10 MHz crystals, we compared the resonance frequency shifts obtained after drop-casting 10 µL of the respective dispersed suspensions and after subsequently exposing the dried films to gas analytes. To determine which configuration yielded the highest response to EG, we examined various concentrations of Cu_2_O and MXene.

**Fig. 4:**
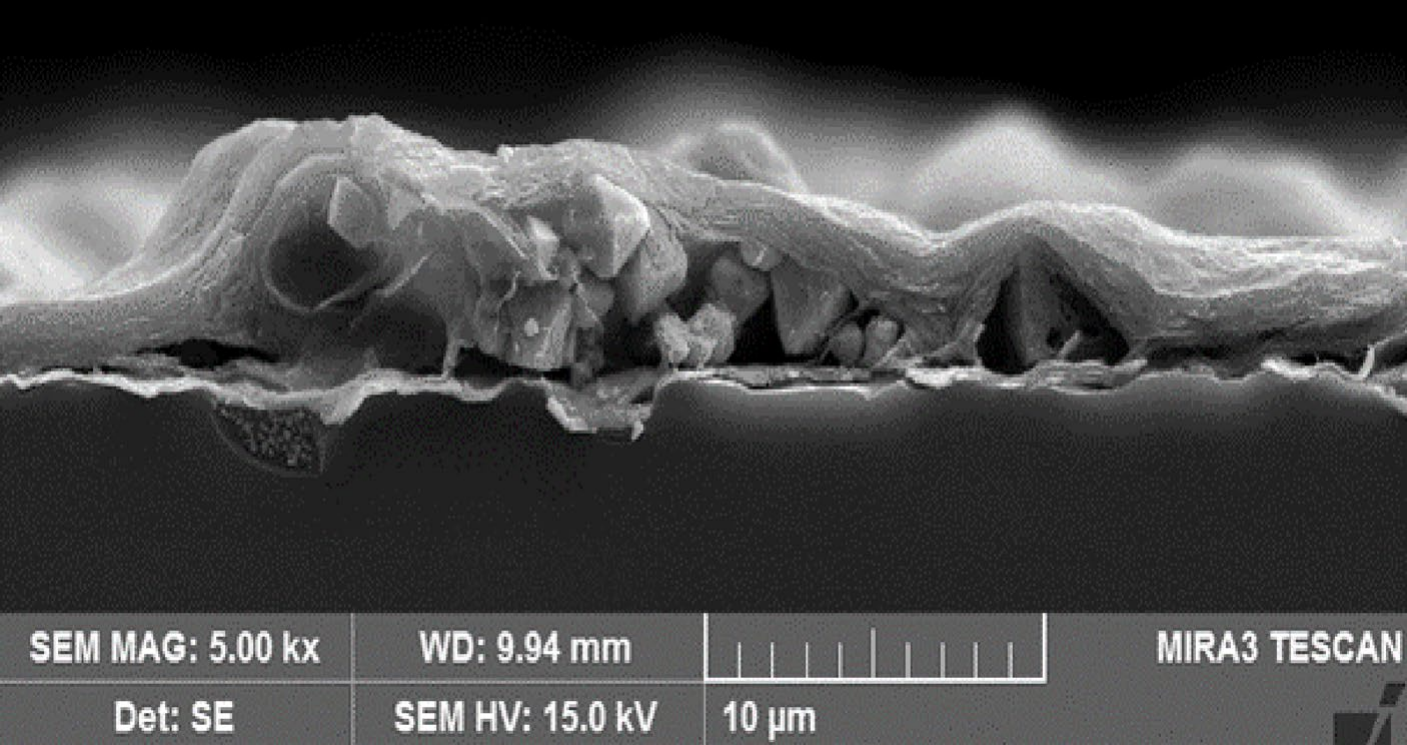
FE-SEM cross-section image of Cu_2_O/MXene mixture sample.

The optimal concentration for single-material Cu_2_O-coated sensors was determined to be 4 mg/mL of cuprous powder dispersed in ethanol, which produced the highest gas response. For mixture samples, the best performance was achieved with 3 mg/mL of Cu_2_O mixed with MXene, resulting in a frequency shift equal to 725 Hz when exposed to 72 ppm EG. SEM analysis (Fig. 4) revealed that in the mixture configuration, Cu_2_O particles were partially buried beneath the MXene layers, thereby limiting their exposure to EG. As a result, the sensing mechanism in these samples is predominantly attributed to the MXene layers.

**Fig. 5:**
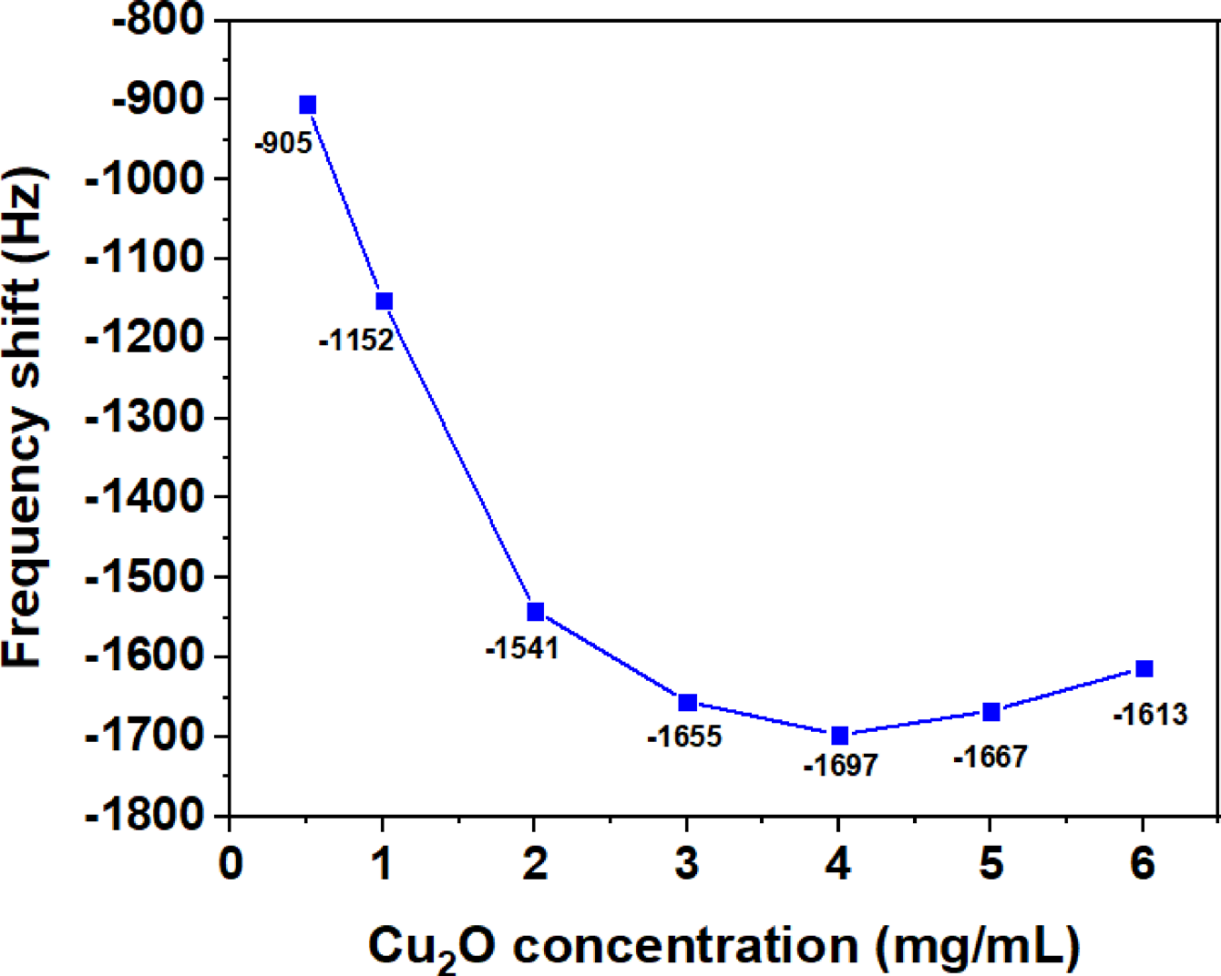
Frequency shift to 72 ppm EG for different Cu_2_O concentrations in the 10 µL drop-casted layer on the MXene-coated quartz crystal.

For the bilayer samples, a 10 µL suspension of Cu_2_O dispersed in ethanol at concentrations varying from 0.5 to 6 mg/mL was drop-cast onto the MXene surface. Fig. 5 shows that at 72 ppm of EG, increasing the Cu_2_O concentrations from 0.5 to 2 mg/mL leads to significant frequency shifts with a steep slope. Between 2 and 4 mg/mL, however, the frequency change exhibits a gentle slope, and above 4 mg/mL the shift reduces, possibly due to excessive coverage of the MXene surface by Cu_2_O. At concentrations of 7 mg/mL and higher, the QCM crystal stops oscillating, likely due to uneven coating distribution or excessive weight of the coating. Thus, the optimal Cu_2_O concentration was determined to be 4 mg/mL.

**Fig. 6:**
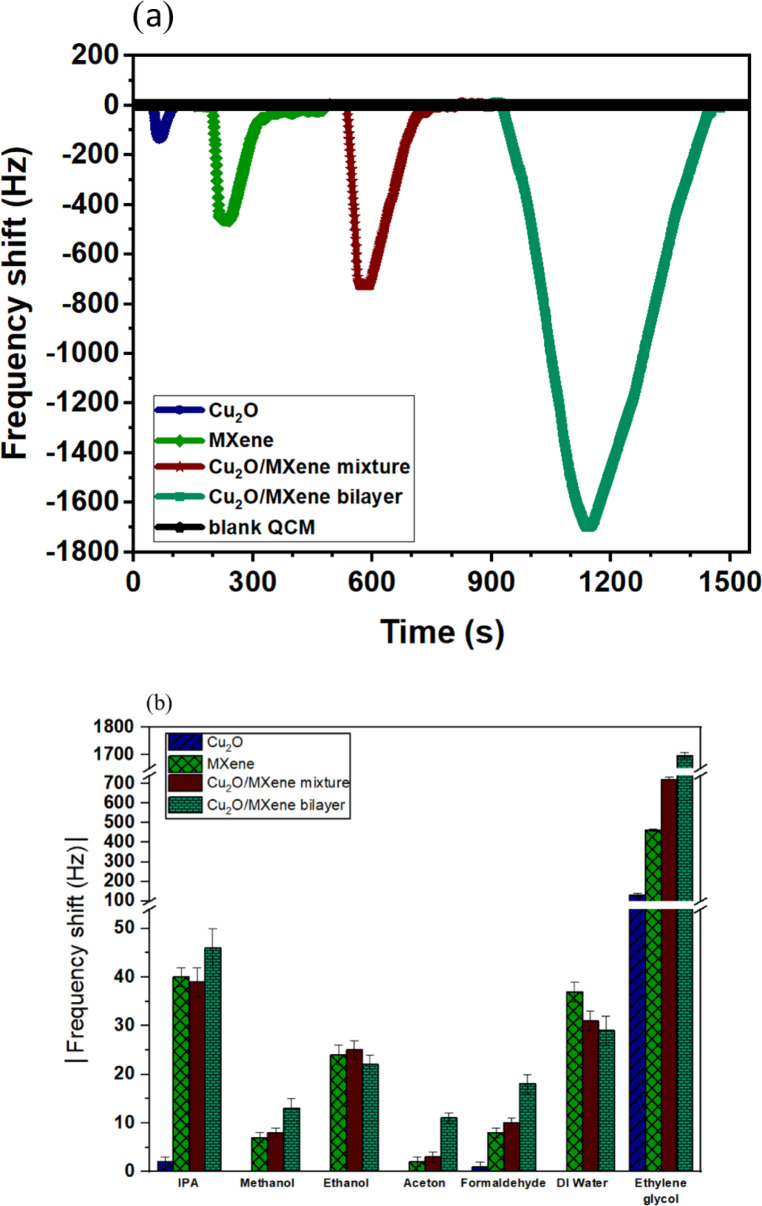
(**a**) Typical response curves of the sensors to EG at 72 ppm; (**b**) Absolute value of the frequency shift of the sensors to different analytes at 72 ppm.

The sensing performance of the Cu_2_O, MXene, and Cu_2_O/MXene films on Quartz Crystal Microbalance (QCM) was assessed across a range of EG concentrations from 375 ppb to 72 ppm. The Cu_2_O/MXene bilayers achieved the highest response (1697 Hz at 72 ppm), significantly outperforming the Cu_2_O-coated QCM electrodes, which showed the lowest response (134 Hz). Fig. 6a depicts the response curves of the sensors to the 72 ppm EG concentration. The selectivity of the Cu_2_O/MXene bilayer sensors was assessed by exposing them to various common volatile chemicals. Fig. 6b presents the frequency shifts of the samples when exposed to isopropyl alcohol (IPA), methanol, ethanol, acetone, formaldehyde, deionized water, and EG, all at a concentration of 72 ppm. For all samples, the highest response belongs to EG compared to the other tested volatile chemicals. It should be noted that the Cu_2_O sensor had a relatively low response (around 134 Hz) to EG, IPA and formaldehyde, and almost no response to the other gases. Whereas the MXene sensors showed a rather considerable response to all gases due to the existence of surface functional groups, they exhibited about ten times more response to EG. The interesting point to note is the synergistic effect of Cu_2_O on MXene film for EG response enhancement (about 3.6 times).

**Fig. 7:**
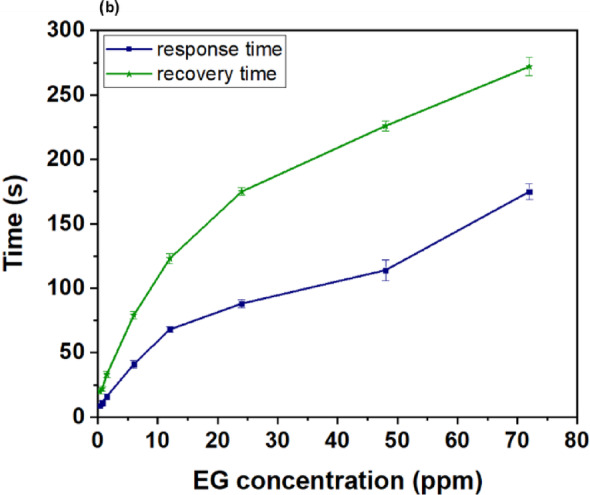
(**a**) Responses of the Cu_2_O/MXene bilayer sensor towards different concentrations of EG; (**b**) Response and recovery time of the Cu_2_O/MXene bilayer sensor towards different concentrations of EG.

As shown in Fig. 7a and its inset, the sensor exhibited a linear response to EG concentrations ranging from 375 ppb to 72 ppm, with a calculated sensitivity of 22.8 Hz/ppm. The Limit of Detection (LOD) was determined to be 381 ppb based on the signal-to-noise ratio methodology. It is worth noting that the blank crystals showed almost no response (Fig. 6a). The LOD of the fabricated QCM gas sensor was calculated, using the data from Fig. 7a, as three times the signal-to-noise ratio using the following formula:

6 $$\:LOD=\frac{3*\sigma\:}{m}$$

Where *LOD* refers to the minimum concentration of an analyte that can be reliably identified, though not necessarily measured precisely. Here, *σ* indicates the standard deviation of the response, reflecting data variability caused by noise or other factors, and *m* represents the slope of the calibration curve, derived from a linear regression analysis of the response versus concentration. This approach yielded a 381 ppb LOD at 25 °C.

Fig. 7b demonstrates the response and recovery times of the Cu_2_O/MXene bilayer. The response time (t_90_) is operationally defined as the time interval required for the sensor output to attain 90% of its equilibrium value upon analyte exposure, whereas the recovery time (t_10_) represents the duration necessary for the signal to decay to 10% of its maximum value following analyte removal. Note that at 6 ppm EG, the sensor achieved a response time of 41 s and a recovery time of 79 s. As can be seen, at low EG concentrations (< 12 ppm), both response and recovery times show steep concentration dependence, while at higher concentrations (> 12 ppm), the trend exhibits a reduced slope.

**Fig. 8:**
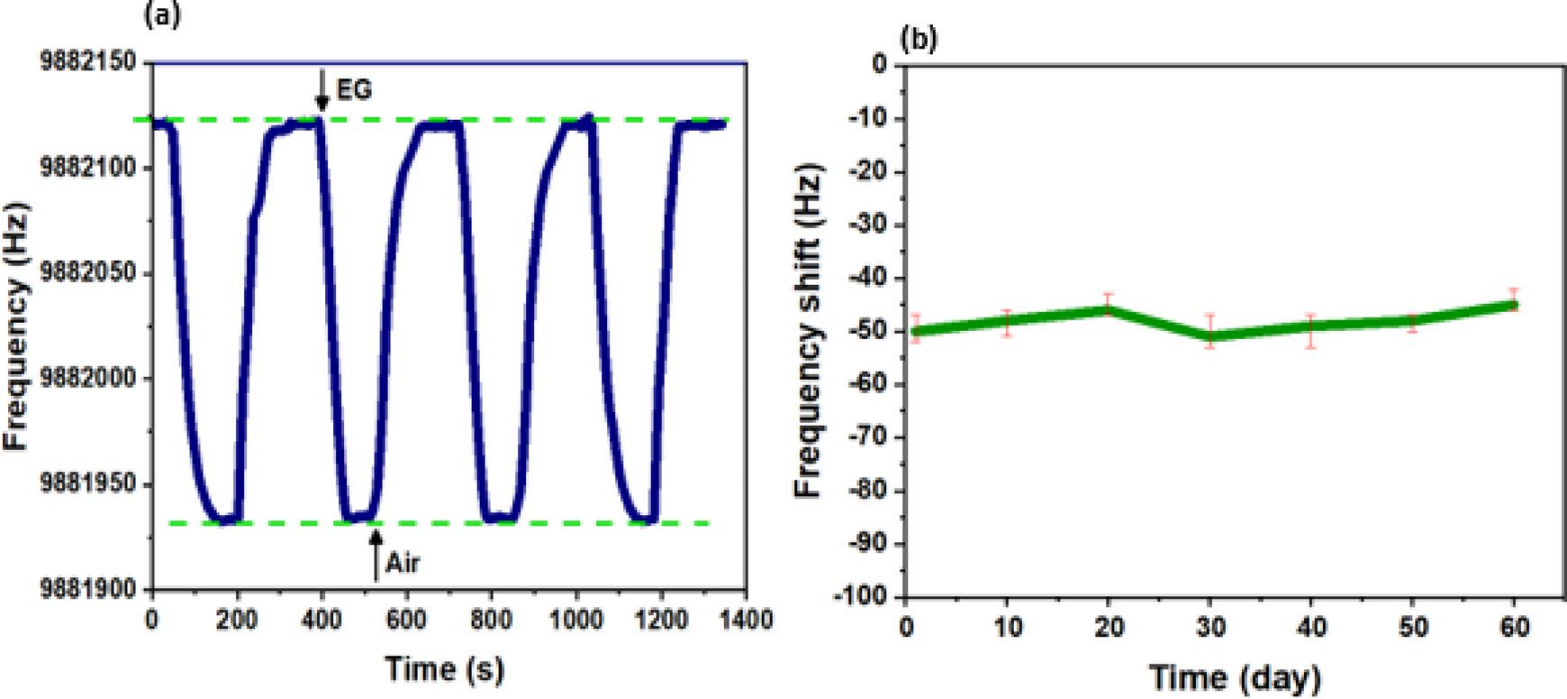
(**a**) Responses of the Cu_2_O/MXene bilayer sensor towards 6 ppm EG; (**b**) Frequency shifts of the Cu_2_O/MXene bilayer sensor towards 750 ppb EG in different days.

The Cu_2_O/MXene bilayer samples presented good recovery for repeating the sensing cycles many times as is shown in Fig. 8a. The stability of the sensors was tested and the sensor’s performance demonstrated excellent repeatability and stability over repeated sensing cycles (Fig. 8a) and maintained consistent frequency shifts over a two-month period (Fig. 8b).

Performance comparisons with previously documented EG sensors emphasize the advantages of the drop-casting method used in this study, highlighting its simplicity, effectiveness, and the reduced requirement for specialized equipment compared to other sensor fabrication techniques. Additionally, our sensor exhibits a notably lower detection threshold (381 ppb) relative to many sensors recently reported in the literature (Table 1). Most significantly, this QCM gas sensor is designed specifically to operate effectively at room temperature (RT), presenting substantial practical advantages for real-world applications.

**Table 1:** Performance of reported EG sensors.

Materials	OT (°C)	Sensor type	LOD	References
2D SnO_2_ nanosheets	130	Resistive sensor	1.37 ppm	^45^
ErFeO_3_ nanofibers	230	Resistive sensor	35 ppb	^46^
CuO–NiO nanotubes	110	Semiconductor sensor	74 ppb	^7^
ZnO/ZnCo_2_O_4_ composite	160	Resistive sensor	1.59 ppm	^47^
ZnO/rGO nanosheets	220	Resistive sensor	1 ppm	^48^
NiO foam@Sn-doped In_2_O_3_ nanowire	125	Resistive sensor	472 ppb	^9^
Cu_2_O/MXene bilayer QCM gas sensor	RT	QCM	381 ppb	This work

### Impact of humidity

Active gas sensors are typically deployed in dynamic ambient environments, which can impact the sensor’s performance^49^. Therefore, it is crucial to study the impact of humidity on the sensor’s performance. We present data for the MXene sample as reference and the Cu_2_O/MXene bilayer which showed the best sensing performance.

**Fig. 9:**
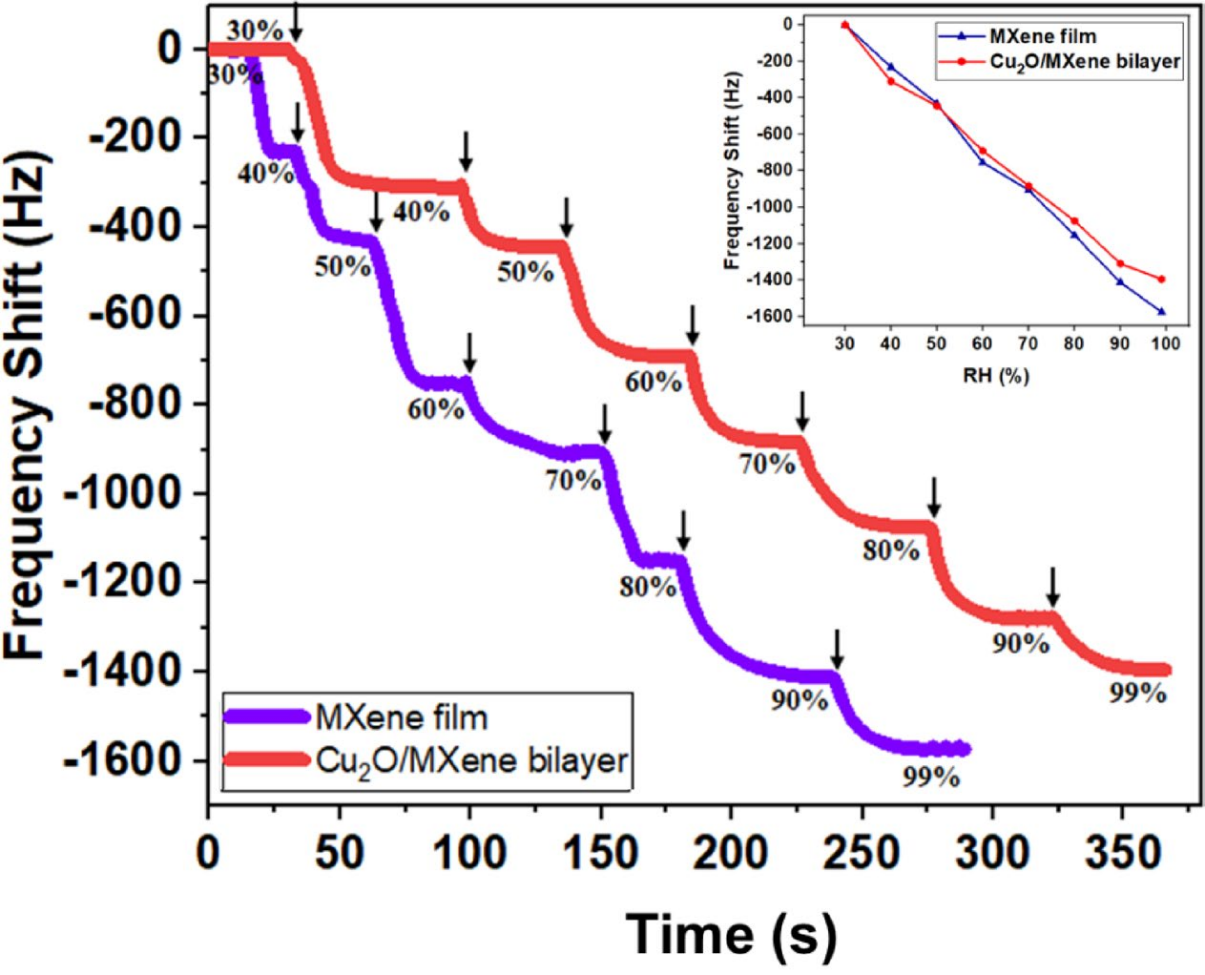
Impact of pure humidity on MXene and Cu_2_O/MXene bilayer QCM sensor. Arrows indicate the times of humidity input.

Fig. 9 and its inset show the frequency change as a function of time at different relative humidity (RH) levels, ranging from 30% (the typical ambient humidity level in our lab) to 99%. The graph clearly indicates that for both the MXene film and the Cu_2_O/MXene bilayer, the sensor’s frequency decreased as relative humidity increased. The frequency dropped almost linearly from 30 to 99% RH, and at higher humidity levels the frequency shifts were slightly more pronounced for the MXene sample than for the bilayer. This suggests that the MXene sample is slightly more sensitive to pure moisture in air than the bilayer.

**Fig. 10:**
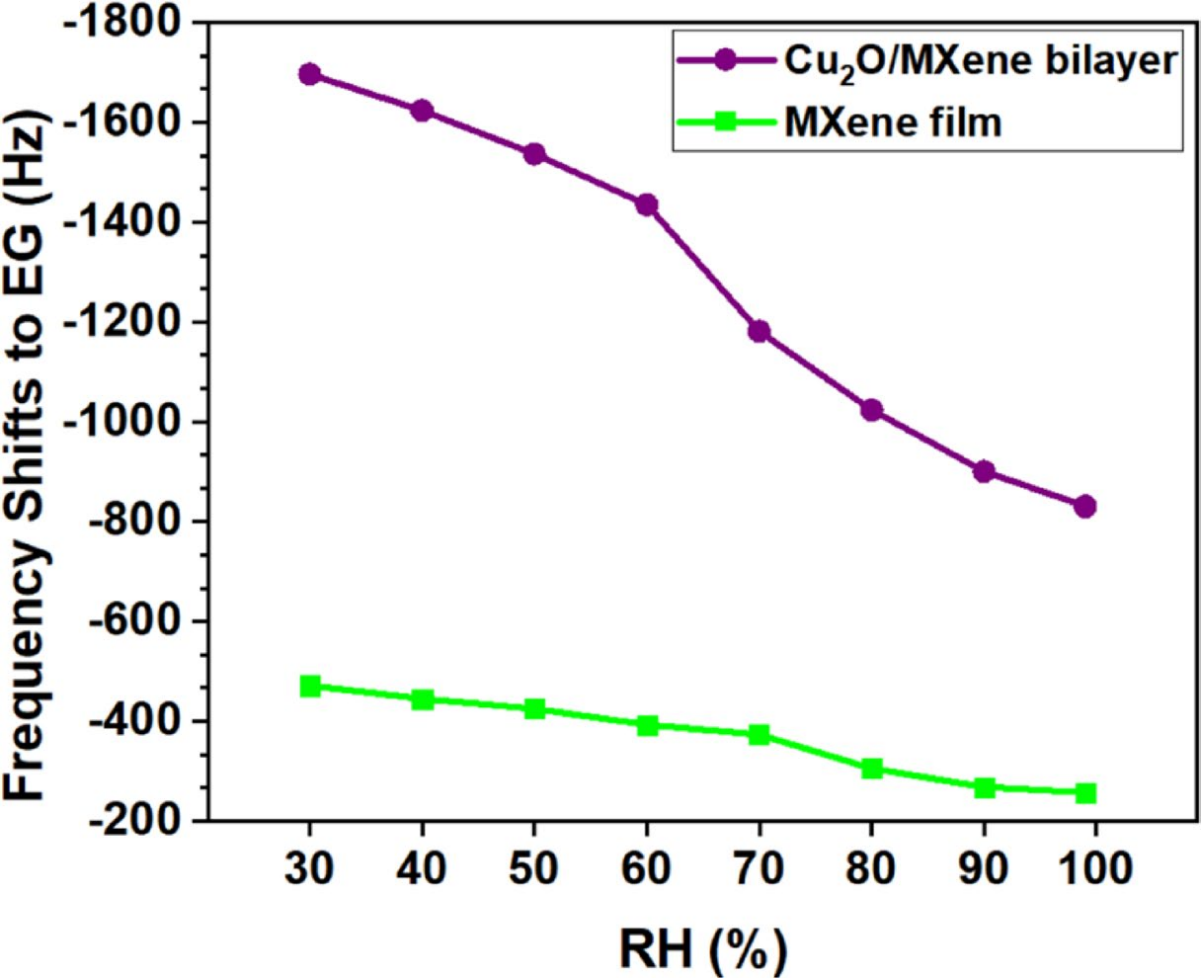
Frequency shifts of MXene and Cu_2_O/MXene bilayer QCM sensor to 72 ppm EG at different relative humidity (RH).

To further study the impact of EG in the presence of humidity, the above sensors were tested by adding 72 ppm EG in air with different relative humidities. The frequency shift versus RH in Fig. 10 indicates less response in high humidity region for both samples (~ 15% response reduction at 60% RH). This can be attributed to the adsorption of lighter water molecules (~ 18 g/mol) onto the Cu_2_O and MXene surfaces through hydrogen bonding, resulting in smaller frequency shifts compared to the adsorption of heavier EG molecules (~ 62 g/mol).

### Gas sensing mechanism

Experimental data indicated significantly higher sensitivity to EG for the Cu_2_O-coated MXene films compared with the individual Cu_2_O, MXene, and MXene-Cu_2_O mixture films (approximately 12.6, 3.6, and 2.34 times greater sensitivity, respectively). This clear enhancement can be attributed to the synergistic effect arising from the addition of Cu_2_O particles onto the MXene films. Specifically, MXene’s 2D structure, characterized by a high surface area and the existence of functional groups such as hydroxyl (-OH), carbonyl (C = O), and fluorine (C-F), provides numerous sites for hydrogen bonding with EG molecules^50^. The incorporation of Cu_2_O particles onto the MXene layer increases the availability of active sites, enhancing the diffusion and adsorption of EG gas molecules (Fig. 12a).

**Fig. 11:**
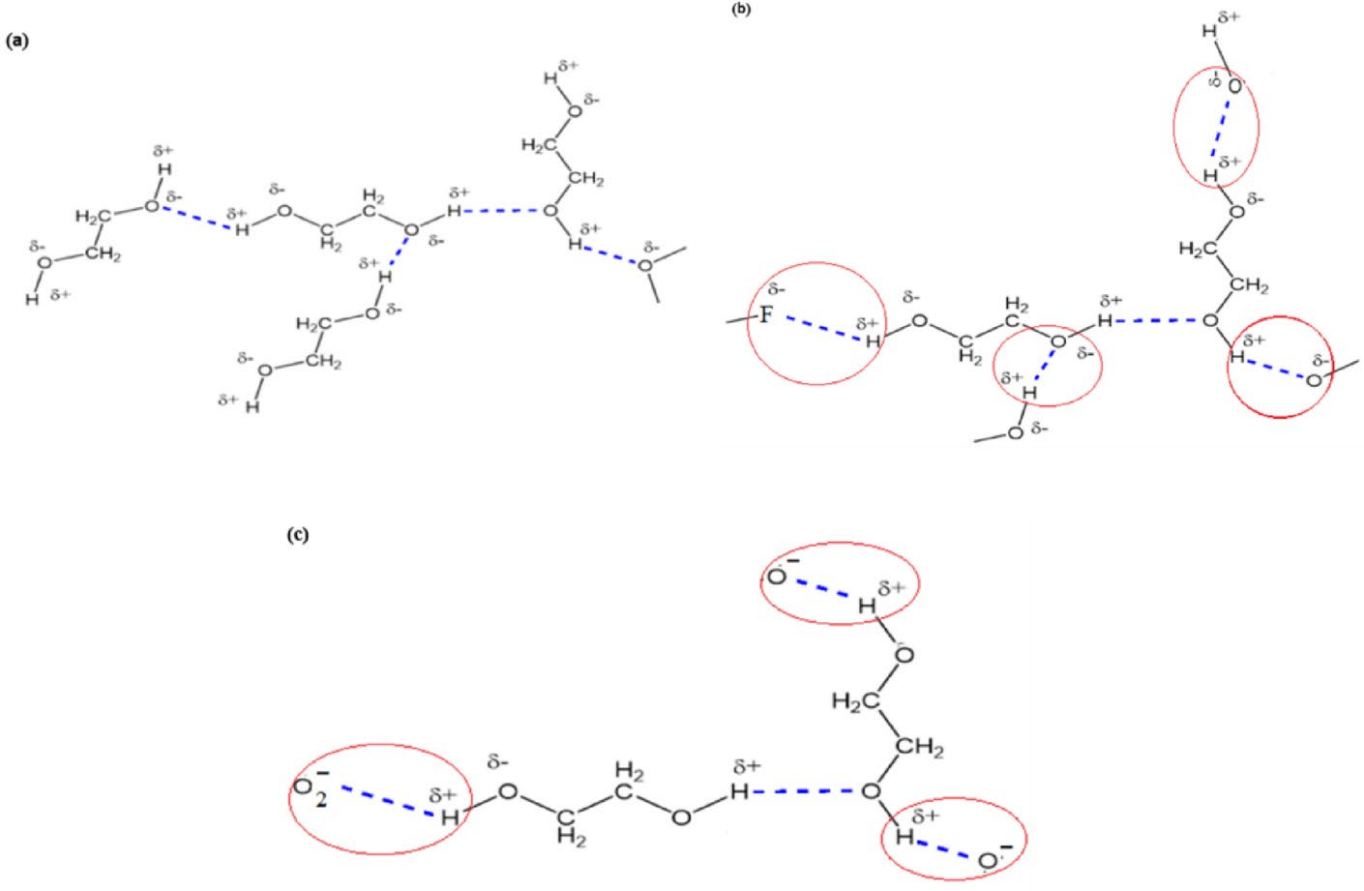
Schematic illustration of hydrogen bonding interactions: (**a**) between ethylene glycol molecules; (**b**) between ethylene glycol molecules with other polar substances; (**c**) between ethylene glycol molecules with surface oxygen groups of Cu_2_O.

EG molecules contain polar O-H groups, in which the higher electronegativity of oxygen compared to hydrogen results in a dipole characterized by partial negative and positive charges (δ^-^, δ^+^) on the oxygen and hydrogen atoms respectively. These dipoles facilitate the creation of hydrogen bonds between EG molecules, and the functional groups present on MXene (Fig. 11a-b). It is important to note that intermolecular hydrogen bonding is a relatively weak interaction, thus considered as physisorption and a nearly complete recovery is observed when the samples are exposed to fresh air.

In the case of Cu_2_O/MXene samples, the following relations can describe the physical reaction that occurs when a Cu_2_O film is exposed to air^51^:

7 $$\:{O}_{2}\left(gas\right)\:\rightleftharpoons\:{O}_{2}\left(ads\right)$$

8 $$\:{O}_{2}\left(ads\right)\:+\:{e}^{-}\rightleftharpoons\:{O}_{2}^{-}\left(ads\right)\;\; (\text{T} < 100 \; ^ {\circ} \text{C})$$

9 $$\:{O}_{2}^{-}\left(ads\right)\:+\:{e}^{-}\rightleftharpoons\:{2O}^{-}\left(ads\right) \;(100\; ^ {\circ} \text{C} < \text{T} < 300\; ^ {\circ} \text{C}, \;\; \text{limited at RT})$$

The adsorption of oxygen ions on the surface and the subsequent formation of a negative surface charge facilitates the hydrogen bonding with the positively charged hydrogen atoms in EG molecules (Fig. 11c).

Since our measurements were conducted at room temperature, the dissociation of oxygen molecules into reactive oxygen ions on the Cu_2_O surface is limited, and physisorbed oxygen molecules predominate^51^.

**Fig. 12:**
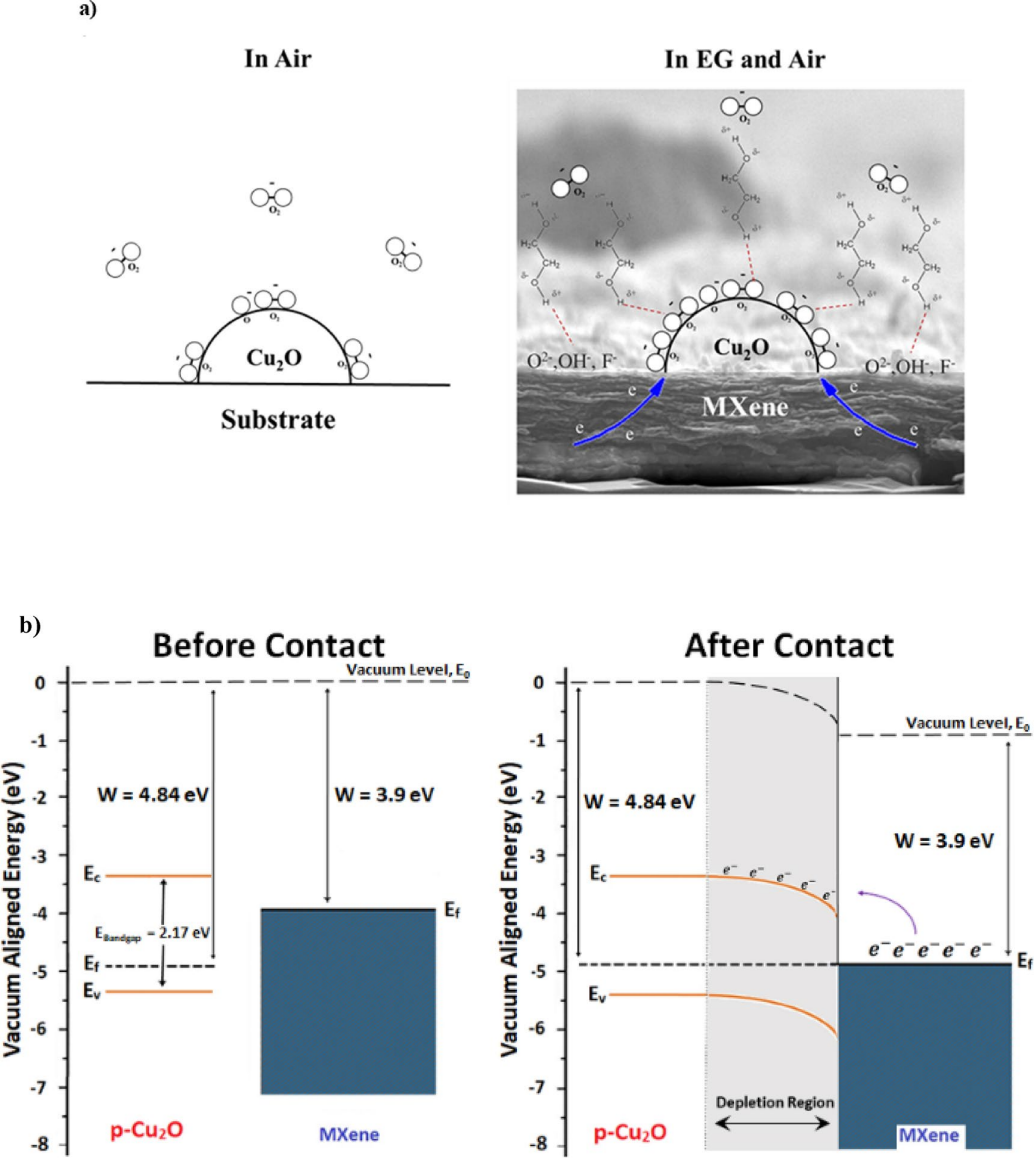
(**a**) Schematic representation of the Cu_2_O/MXene surface in air and after EG introduction, (**b**) energy band-diagram of Cu_2_O and MXene before and after contact.

To describe synergetic effect, we assumed the Cu_2_O particle in contact with MXene, forms a semiconductor-metal interface in which electrons can transfer from MXene (with a negative zeta potential and electrically conductive properties) to the p-type semiconductor Cu_2_O particles. This electron transfer leads to energy band bending and the formation of a Schottky junction, creating a charge depletion region in the Cu_2_O particles (Fig. 12b). With MXene’s work function being lower (3.9 eV) than that of Cu_2_O (4.84 eV), electron transfer occurs^52,53^. Although charge transfer is a well-known mechanism in chemiresistive sensors, the sensing response in Quartz Crystal Microbalance (QCM) devices is fundamentally related to the mass loading and viscoelastic changes on the sensor surface, which directly affect the resonant frequency of the quartz crystal. In the case of the Cu_2_O/MXene bilayer, charge transfer results more adsorbed oxygen molecules as is shown in Fig. 12a, thereby increasing the mass loading on the QCM surface. This synergistic effect explains the improved sensitivity observed in the bilayer sensor. The synergistic effect between Cu_2_O and MXene at their interface has been observed on CO_2_ reduction and antibacterial applications^54,55^. This effect arises from the favorable interaction, leading to improved charge transfer, increased stability, and enhanced catalytic activity. It is worth noting that calculations indicate that the width of the depletion region is in the order of the Cu_2_O particle size.

The selectivity of the bilayer sensors, demonstrated in Fig. 6b, highlights their pronounced response to EG relative to the other shown tested volatile chemicals. This specificity is mainly due to the molecular structure of EG, which contains two hydroxyl groups, allowing for stronger interactions compared to molecules like isopropyl alcohol (IPA) and ethanol, which contain fewer hydroxyl groups. These dual hydroxyl (-OH) groups in EG facilitate multiple hydrogen bonds with the abundant functional groups on the MXene surface (such as -OH, C = O, and C-F) as well as the surface oxygen sites on Cu_2_O particles, enhancing the adsorption affinity and thereby the sensor’s selective response. In addition, the relatively higher molecular weight of EG compared to the other tested gases contributes to a larger mass loading effect on the Quartz Crystal Microbalance sensor.

## Conclusion

We have developed a Cu_2_O/MXene film-based Quartz Crystal Microbalance (QCM) gas sensor that demonstrates high sensitivity and selectivity for detecting EG at room temperature. Among various prepared samples with different film configurations, the bilayer structure exhibited superior response compared to the single-layer Cu_2_O, MXene, or their mixture coated quartz crystals. The impact of relative humidity becomes significant above 60%, indicating the necessity of humidity compensation for the practical sensor deployment. The sensing mechanism primarily involves physisorption and relies on hydrogen bonding between the functional groups on MXene’s expansive surface and the dual hydroxyl groups of EG molecules. Additionally, the formation of a depletion region at the Schottky junction between the conductive MXene and the p-type Cu_2_O particles significantly enhances gas sensitivity through charge transfer to the oxide surface, synergistically improving EG adsorption. The advanced sensitivity and robust performance of this sensor hold significant implications for enhancing environmental monitoring, industrial safety applications, and public health protection.

## Data Availability

The datasets used and/or analyzed during the current study available from the corresponding author on reasonable request.
